# Anion inhibition studies of a beta carbonic anhydrase from the malaria mosquito *Anopheles gambiae*

**DOI:** 10.1080/14756366.2017.1421182

**Published:** 2018-01-11

**Authors:** Daniela Vullo, Leo Syrjänen, Marianne Kuuslahti, Seppo Parkkila, Claudiu T. Supuran

**Affiliations:** a Dipartimento di Chimica, Laboratorio di Chimica Bioinorganica, Università degli Studi di Firenze, Sesto Fiorentino (Firenze), Italy;; b Faculty of Medicine and Life Sciences, University of Tampere, Tampere, Finland;; c Fimlab Laboratories Ltd, Tampere, Finland;; d Neurofarba Dipartment, Sezione di Scienza Farmaceutiche e Nutraceutiche, Università degli Studi di Firenze, Sesto Fiorentino (Firenze), Italy

**Keywords:** Carbonic anhydrase, anion, *Anopheles gambiae*, inhibitor, malaria

## Abstract

An anion inhibition study of the β-class carbonic anhydrase, AgaCA, from the malaria mosquito *Anopheles gambiae* is reported. A series of simple as well as complex inorganic anions, and small molecules known to interact with CAs were included in the study. Bromide, iodide, bisulphite, perchlorate, perrhenate, perruthenate, and peroxydisulphate were ineffective AgaCA inhibitors, with K_I_s > 200 mM. Fluoride, chloride, cyanate, thiocyanate, cyanide, bicarbonate, carbonate, nitrite, nitrate, sulphate, stannate, selenate, tellurate, diphosphate, divanadate, tetraborate, selenocyanide, and trithiocarbonate showed K_I_s in the range of 1.80–9.46 mM, whereas *N,N*-diethyldithiocarbamate was a submillimolar AgaCA inhibitor (K_I_ of 0.65 mM). The most effective AgaCA inhibitors were sulphamide, sulphamic acid, phenylboronic acid and phenylarsonic acid, with inhibition constants in the range of 21–84 µM. The control of insect vectors responsible of the transmission of many protozoan diseases is rather difficult nowadays, and finding agents which can interfere with these processes, as the enzyme inhibitors investigated here, may arrest the spread of these diseases worldwide.

## 1. Introduction

Malaria is a tropical disease transmitted mainly by mosquitoes of the genus *Anopheles*. The disease itself is caused by parasites of the genus *Plasmodium*, of which five species affect human red blood cells: *P. falciparum, P. ovale, P. vivax, P. knowlesi and P. malariae*. Of these *P. falciparum* causes the most severe infection. Malaria causes non-specific symptoms of which most common are fever and headache, and in severe cases the disease may lead to death^1^.

Carbonic anhydrases (CAs, EC 4.2.1.1) are enzymes that catalyse the reversible hydration reaction of carbon dioxide according to the following reaction^2^: CO_2_ + H_2_O ↔ HCO_3_^−^ + H^+^. CAs are typically zinc-containing metalloenzymes, but the ζ form uses cadmium or zinc (in an inter-exchangeable manner), as an alternative metal cofactor^3^^,^^4^. In addition, γ-CAs may contain iron(II) within the active site, at least in some anaerobic *Archaea*^5^^,^^6^. The reaction catalysed by CAs is crucial in the regulation of acid–base balance in organisms. Additionally, this reaction participates in removing carbon dioxide out of tissues, takes part in biosynthetic reactions such as gluconeogenesis and ureagenesis, and is involved in many other physiological processes as well^2^. Seven classes of carbonic anhydrases have been identified: the α, β, γ, δ, ζ, η and θ-CAs^7^. Of these, β-CAs appear to be the family with the widest distribution^7^. They have been described from various groups of organisms including the *Bacteria* and *Archaea* domains as well as in all species of plants and some fungi among *Eukarya*^8^. In addition, our previous studies suggested the widespread occurrence of at least one single-copy of a β-CA gene among animal species distinct from chordates^9^.

β-CAs were first reported from two invertebrate species: the fruit fly *Drosophila melanogaster* and the worm *Caenorhabditis elegans*^9^^,^^10^. Additionally, a β-CA was characterised from the unicellular parasite *Leishmania donovani chagasi* which is one of the causative agents for visceral leishmaniasis^11^. Previously two α-CAs have been characterised from the mosquito *Anopheles gambiae*^12^^,^^13^. Despite the recent finding that *Leishmania* parasites encode β-CAs, some protozoan parasites possess only α- or η-CAs. For example, *P. falciparum* seems to encode only the η-CAs^14^. The exact expression pattern of β-CAs in protozoan organisms is currently unclear, even though a previous report suggested the presence of this enzyme family in a number of protozoans and metazoans^14^c. Recently, α-CA was characterised from unicellular protozoa responsible of Chagas’ disease, *Trypanosoma cruzi*^15^.

β-CAs are not present in vertebrates, which may lead to the design of β-CA-specific inhibitors that could be used against invertebrate pathogens and pathogen vectors with minimal side effects on vertebrate species. However, no such inhibitors have been reported so far. We performed sequence searches in the genomes of different pathogen vectors and found out that *Anopheles gambiae* encodes for one β-CA in addition to several α-CAs. The aim of this study was to characterise further the β-CA, AgaCA, from malaria mosquito *Anopheles gambiae*, which is one of the most important malaria vector organisms^1^. Kinetic and anion inhibition studies with a large set of inhibitors were carried out to characterise its catalytic activity and inhibition profile, considering the fact that we have reported earlier only the sulphonamide inhibition profile of this enzyme^16^.

## 2. Materials and methods

### 2.1. Construction of β-CA fusion protein

*Anopheles gambiae* cDNA was obtained from Professor Michael Lehane (Liverpool School of Tropical Medicine, UK). The β-CA gene was retrieved from NCBI protein databases using Blast^17^, http://blast.ncbi.nlm.nih.gov/Blast.cgi. The full-length β-CA gene was identified and amplified from cDNA by PCR using Phusion^TM^ Hot Start High Fidelity DNA Polymerase (Finnzymes, Espoo, Finland). The detailed PCR method has been described previously by our group^16^. The PCR product band was separated from the gel and dissolved using Illustra™ GFX PCR DNA and GEL Band Purification Kit (GE Healthcare Life Sciences, Buckinghamshire, UK). Validity of the PCR product was verified by sequencing.

For recombinant protein production, the β-CA gene was constructed and cloned into the pFastBac1TM vector. The forward primer used in the initial amplification of the β-CA gene was 5′-CGCGGATCCATGGAGCGTATATTGCGAGGC-3′ (F2), and the reverse primer was 5′-GCCCTCGAGTTAATGGTGGTGATGGTGGTGGGAACC-ACGGGGCACCAGCGAATAGTATCGCCGTACCTC-3′ (R2). The latter primer contains nucleotide repeats to create the C-terminal polyhistidine tag with six histidines. In addition, the forward primer contained the restriction site for BamHI and the reverse primer for XhoI. The reverse primer also contained the nucleotide sequence encoding thrombin cleavage site. The PCR program was as follows: 98 °C for 30 s; then 35 cycles of 98 °C for 10 s, 62 °C for 15 s, and 62 °C for 30 s, and finally 72 °C for 5 min.

The PCR product was run on an agarose gel, and the obtained band was purified. pFastBac^TM^1 plasmid (Invitrogen, Carlsbad, CA) and the PCR product were digested at +37 °C overnight with BamHI and XhoI restriction enzymes (New England Biolabs, Ipswich, MA). The digested plasmid and PCR product containing full-length recombinant *A. gambiae* β-CA gene were purified and then ligated overnight at +4 °C using T4 DNA ligase (New England Biolabs). The ligated product was transformed into TOP10 bacteria (Invitrogen, Helsinki, Finland). Overnight cultures (8 ml) were made from these colonies, and plasmids were purified using a QIAprep Spin Miniprep Kit™ (Qiagen, Hilden, Germany). The construction of baculoviral genomes encoding the recombinant proteins has been described previously^18^.

### 2.2. Production of *A. gambiae* β-CA

The Sf9 insect cells were grown in Insect-Xpress protein-free cell culture medium (Lonza, Verviers, Belgium) in an orbital shaker at 27 °C (125 rpm) for 3 d after infection. Protein purification was performed after centrifugation (5000 × *g*, 20 °C, 8 min) from the supernatant. Purification was performed using the Protino® Ni-NTA Agarose (from Macherey-Nagel, Munich, Germany) under native binding conditions with wash and elution buffers made according to the manufacturer's instructions. The purification procedure per 400 ml of insect cell medium was as follows: 3 L of native binding buffer (50 mM NaH_2_PO_4_, 500 mM NaCl, pH 8.0) and 8 ml of the nickel-chelating agarose were added to the medium, and the His-tagged protein was then allowed to bind to the resin on a magnetic stirrer at 25 °C for 3 h. The resin was washed with 40 + 20 ml of washing buffer (50 mM NaH_2_PO_4_, 500 mM NaCl, 20 mM imidazole pH 8.0). The protein was then eluted with elution buffer (50 mM NaH_2_PO_4_, 500 mM NaCl, 250 mM imidazole, pH 8.0). After this, the protein was transferred to 50 mM Tris-HCl, pH 7.5. To remove the His tag, the recombinant protein was treated with 150 μL of resin-coupled thrombin (Thrombin CleanCleave KIT™, Sigma, Milan, Italy) per 1 mg of protein with gentle shaking at +20 °C overnight, according to the manufacturer's instructions. Protein concentration was determined using the DC Protein Assay™ (Bio-Rad, Berlin, Germany) with three different dilutions.

### 2.3. CA activity measurements and inhibition studies

An Applied Photophysics stopped-flow instrument has been used for assaying the CA catalysed CO_2_ hydration activity^19^. Phenol red (at a concentration of 0.2 mM) has been used as indicator, working at the absorbance maximum of 557 nm, with 20 mM Hepes (pH 7.6) or 20 mM TRIS (pH 8.3) as buffers, and 20 mM NaClO_4_ (for maintaining constant the ionic strength). Perchlorate is not inhibiting the enzyme at concentrations up to 100 mM, data not shown, as for many other CAs investigated earlier by our group^20^), following the initial rates of the CA-catalysed CO_2_ hydration reaction for a period of 10–100 s^19^. The CO_2_ concentrations ranged from 1.7 to 17 mM for the determination of the kinetic parameters and inhibition constants. For each inhibitor at least six traces of the initial 5–10% of the reaction have been used for determining the initial velocity. The uncatalysed rates were determined in the same manner and subtracted from the total observed rates. Stock solutions of inhibitors (10 mM) were prepared in distilled-deionised water and dilutions up to 0.01 nM were done thereafter with the assay buffer. Inhibitor and enzyme solutions were preincubated together for 15 min at room temperature prior to assay, in order to allow for the formation of the E-I complex. The inhibition constants were obtained by non-linear least-squares methods using PRISM 3 and the Cheng–Prusoff equation, as reported earlier^21–23^, and represent the mean from at least three different determinations. All CA isoforms were recombinant ones obtained in house as reported earlier^9^^,^^16^. The concentration of AgaCA used in the experiments reported in the paper was of 13.2 nM.

## 3. Results and discussion

As shown in the introduction, there exists only few studies on insect CAs. Apart our initial reports^9^^,^^16^ on the presence of a β-CA in *Drosophila melanogaster* and *Anopheles gambiae*, some sulphonamide and dithiocarbamate studies were reported for the inhibition of the first enzyme^24^, but no other inhibition studies (except the sulphonamide ones)^16^ are available for AgaCA. It should be mentioned that recently a CA was also reported and its activity/inhibition investigated from another insect species, the honey bee *Apis mellifera*^25^. In this paper, we report the first extensive anion inhibition study of the β-CA from *Anopheles gambiae*, AgaCA, with a large series of simple and complex anions.

In the previous work^16^, we observed that AgaCA has a significant catalytic activity for the physiologic, CO_2_ hydration reaction to bicarbonate and protons, with the kinetic parameters shown in Table 1. AgaCA has a catalytic activity which is similar to that of the human cytosolic isoform hCA I, and is also inhibited quite effectively by the sulphonamide. The widely clinically used compound, acetazolamide (5-acetamido-1,3,4-thiadiazole-2-sulphonamide), showed an inhibition constant of 27.3 nM (Table 1).

**Table 1.** Kinetic parameters for the CO_2_ hydration reaction catalysed by the human cytosolic isozymes hCA I and II (α-class CAs) and the β-CAs from *Drosophila melanogaster* (DmBCA) and *Anopheles gambiae* (AgaCA) measured at 20 °C, pH 7.6 in 20 mM HEPES buffer (for hCA I and II) and 20 °C, pH 8.3 in 20 mM TRIS buffer (for the β-CAs), in the presence 20 mM NaClO_4_ (for maintaining constant ionic strength). Inhibition data with the clinically used sulphonamide acetazolamide (5-acetamido-1,3,4-thiadiazole-2-sulphonamide) are also provided.

Enzyme	Activity level	Class	k_cat_ (s^−1^)	k_cat_/K_m_ (M^−1^× s^−1^)	K_I_ (acetazolamide) (nM)
hCA I^a^	Moderate	α	2.0 × 10^5^	5.0 × 10^7^	250
hCA II^a^	Very high	α	1.4 × 10^6^	1.5 × 10^8^	12
DmBCA^b^	High	β	9.5 × 10^5^	1.1 × 10^8^	516
AgaCA^c^	Moderate	β	7.2 × 10^5^	5.6 × 10^7^	27.3

a From ref.^20a^.

b From ref.^9^.

c From ref.^16^.

Inorganic anions constitute an important class of CA inhibitors (CAIs)^20^. Both inorganic, complexing anions and more complex anions were investigated for their interaction with a large number of enzymes belonging to all CA families^20^. Such studies may lead to the discovery of novel classes of pharmacologically relevant CAIs: indeed, the dithiocarbamates were discovered, considering the simple anion trithiocarbonate (CS_3_^2−^) as an inhibitor, and showed significant *in vitro* and *in vivo* activities in pathologies related to CA dysregulation, such as glaucoma^26^.

In Table 2, the inhibition of AgaCA with a panel of such anions is shown. Inhibition data for the widespread cytosolic isoforms hCA I and II, as well as for the enzyme from *D. melanogaster*, are also shown, for comparison reasons. The following may be noted from the inhibition data of Table 2:

(i) Anions with low propensity for inhibiting AgaCA were bromide, iodide, bisulphite, perchlorate, perrhenate, perruthenate, and peroxydisulphate, which showed K_I_s > 200 mM. Whereas perchlorate is generally the anion with less affinity for metal ions in solution and metalloenzyme (in fact it does not inhibit significantly any CA investigated so far)^20^, the data for the heavy halogenides and bisulphite are rather surprising, considering the fact that iodide and bromide are rather effective hCA I and DmBCA inhibitors (Table 2). Bisulphite is a weak hCA I and II inhibitor but it is more effective as a DmBCA inhibitor.

**Table 2.** Inhibition constants of anionic inhibitors against isozymes hCA I, II (α-CA class), and DmBCA (*D. melanogaster*) and AgaCA (*A. gambiae*) for the CO_2_ hydration reaction, at 20 °C^19^.

	K_I_ [mM]^e^				
Inhibitor	hCA I^a^	hCA II^a^		DmBCA^b^	AgaCA^c^
F^−^	>300	>300		0.80	9.42
Cl^−^	6	200		0.97	8.74
Br^−^	4	63		1.04	>200
I^−^	0.3	26		1.18	>200
CNO^−^	0.0007	0.03		0.73	9.46
SCN^−^	0.2	1.6		1.28	6.41
CN^−^	0.0005	0.02		0.67	8.34
N_3_^−^	0.0012	1.5		1.12	12.40
HCO_3_^−^	12	85		26.90	4.34
CO_3_^2−^	15	73		0.86	9.25
NO_3_^−^	7	35		43.74	6.50
NO_2_^−^	8.4	63		28.60	4.55
HS^−^	0.0006	0.04		1.01	25.10
HSO_3_^−^	18	89		1.29	>200
SO_4_^2−^	63	>200		1.36	9.03
ClO_4_^−^	>200	>200		>200	>200
SnO_3_^2−^	0.57	0.83		nt	1.80
SeO_4_^2−^	118	112		nt	9.41
TeO_4_^2−^	0.66	0.92		nt	4.96
P_2_O_7_^4−^	25.8	48.5		nt	8.52
V_2_O_7_^4−^	0.54	0.57		nt	7.98
B_4_O_7_^2−^	0.64	0.95		nt	7.95
ReO^4−^	0.11	0.75		nt	>200
RuO^4−^	0.10	0.69		nt	>200
S_2_O_8_^2−^	0.11	0.084		nt	>200
SeCN^−^	0.0085	0.086		nt	8.68
CS_3_^2−^	0.0087	0.0088		nt	8.19
Et_2_NCS_2_^−^	0.79	3.1		nt	0.65
H_2_NSO_2_NH_2_	0.31	1.13		0.15	0.054
H_2_NSO_3_H^d^	0.021	0.39		2.45	0.021
Ph-B(OH)_2_	58.6	23.1		22.39	0.047
Ph-AsO_3_H_2_^d^	31.7	49.2		32.60	0.084

a From ref.^20a^.

b From ref.^9^.

c This work.

d As sodium salt; nt: not tested.

e Errors in the range of 5–10% of the shown data, from three different assays, by a CO_2_ hydration stopped-flow assay^19^.

(ii) Azide and hydrogensulphide, anions which show a high affinity for many metal ions^20^, were rather weak AgaCA inhibitors, with K_I_s of 12.4–25.1 mM. They were much more effective as DmBCA inhibitors and are micromolar hCA I inhibitors (Table 2). Thus, there are significant differences in the affinity of these inhibitors for various CAs, with the mosquito enzyme definitely less sensitive to these inhibitors compared to other insect or human CAs.

(iii) Most of the investigated anions showed inhibition constants in the range of 1.80–9.46 mM, being thus weak CAIs, but normally this is the range in which most simple/complex anions interact with most CAs^20^. They include fluoride, chloride, cyanate, thiocyanate, cyanide, bicarbonate, carbonate, nitrite, nitrate, sulphate, stannate, selenate, tellurate, diphosphate, divanadate, tetraborate, selenocyanide, and trithiocarbonate (Table 2). It should be observed that this series includes both anions with a high affinity for complexing metal ions (such as cyanate, thiocyanate, and cyanide) as well as anions with lower affinity for cations, such as nitrite, nitrate, and sulphate. It is interesting to note that for the halogenides, those incorporating light elements (F, Cl) were more effective than the halogenides incorporating heavy elements, which is opposite to the inhibitory effects observed with these anions against hCA I and II (Table 2). Bicarbonate was two times better as a AgaCA inhibitor compared with carbonate, whereas sulphate, which is a weak hCA I and II inhibitor, showed a <10 mM activity against AgaCA.

(iii) *N,N*-Diethyldithiocarbamate was a submillimolar AgaCA inhibitor, with a K_I_ of 0.65 mM, being thus much more effective than trithiocarbonate (K_I_ of 8.19 mM) from which it is derived.

(iv) The most effective AgaCA inhibitors were sulphamide, sulphamic acid, phenylboronic acid, and phenylarsonic acid, with inhibition constants in the range of 21–84 µM. These compounds are known to act as efficient CAIs against many CAs and were used as leads to obtain potent inhibitors, some of which inhibit these enzymes in the low nanomolar range^27^. Indeed, these simple molecules incorporate zinc-binding functions of the sulphonamide, sulphamide, sulphamate, boronic acid, etc., which have been extensively employed to design highly effective CAIs^27^.

## 4. Conclusions

We report here an anion inhibition study of the β-class CA, AgaCA, from the mosquito *Anopheles gambiae*, the vector responsible of malaria transmission. A series of simple as well as complex inorganic anions, together with small molecules known to interact with CAs were included in the study. Bromide, iodide, bisulphite, perchlorate, perrhenate, perruthenate, and peroxydisulphate were ineffective AgaCA inhibitors, with K_I_s > 200 mM. Fluoride, chloride, cyanate, thiocyanate, cyanide, bicarbonate, carbonate, nitrite, nitrate, sulphate, stannate, selenate, tellurate, diphosphate, divanadate, tetraborate, selenocyanide, and trithiocarbonate showed K_I_s in the range of 1.80–9.46 mM, whereas *N,N*-diethyldithiocarbamate was a submillimolar AgaCA inhibitor, with a K_I_ of 0.65 mM. The most effective AgaCA inhibitors were sulphamide, sulphamic acid, phenylboronic acid, and phenylarsonic acid, with inhibition constants in the range of 21–84 µM. The control of insect vectors responsible of the transmission of many protozoan diseases is rather difficult nowadays, and finding agents which can interfere with these processes, as the enzyme inhibitors investigated here, may arrest the spread of these diseases worldwide.

## References

[1] Whitty CJ, Chiodini PL, Lalloo DG. Investigation and treatment of imported malaria in non-endemic countries. BMJ 2013;346:f2900. 23693055 10.1136/bmj.f2900

[2] a) Sly WS, Hu PY. Human carbonic anhydrases and carbonic anhydrase deficiencies. Annu Rev Biochem 1995;64:375–401. 7574487 10.1146/annurev.bi.64.070195.002111

[3] a) Xu Y, Feng L, Jeffrey PD, et al Structure and metal exchange in the cadmium carbonic anhydrase of marine diatoms. Nature 2008;452:56–61. 18322527 10.1038/nature06636

[4] a) Lane TW, Saito MA, George GN, et al Biochemistry: a cadmium enzyme from a marine diatom. Nature 2005;435:42. 15875011 10.1038/435042a

[5] a) Macauley SR, Zimmerman SA, Apolinario EE, et al The archetype gamma-class carbonic anhydrase (Cam) contains iron when synthesized in vivo. Biochemistry 2009;48:817–9. 19187031 10.1021/bi802246s

[6] a) Tripp BC, Bell CB, Cruz F, et al A role for iron in an ancient carbonic anhydrase. J Biol Chem 2004;279:6683–7. 14662760 10.1074/jbc.M311648200

[7] a) Capasso C, Supuran CT. An overview of the alpha-, beta-and gamma-carbonic anhydrases from Bacteria: can bacterial carbonic anhydrases shed new light on evolution of bacteria? J Enzyme Inhib Med Chem 2015;30:325–32. 24766661 10.3109/14756366.2014.910202

[8] a) Supuran CT. Structure and function of carbonic anhydrases. Biochem J 2016;473:2023–32. 27407171 10.1042/BCJ20160115

[9] Syrjanen L, Tolvanen M, Hilvo M, et al Characterization of the first beta-class carbonic anhydrase from an arthropod (*Drosophila melanogaster*) and phylogenetic analysis of beta-class carbonic anhydrases in invertebrates. BMC Biochem 2010;11:28. 20659325 10.1186/1471-2091-11-28 PMC2918522

[10] Fasseas MK, Tsikou D, Flemetakis E, Katinakis P. Molecular and biochemical analysis of the beta class carbonic anhydrases in *Caenorhabditis elegans*. Mol Biol Rep 2010;37:2941–50. 19816790 10.1007/s11033-009-9857-z

[11] Syrjanen L, Vermelho AB, de Almeida Rodrigues I, et al Cloning, characterization and inhibition studies of a beta carbonic anhydrase from Leishmania donovani chagasi, the protozoan parasite responsible of leishmaniasis. J Med Chem 2013;56:7372–81. 23977960 10.1021/jm400939k

[12] Seron TJ, Hill J, Linser PJ. A GPI-linked carbonic anhydrase expressed in the larval mosquito midgut. J Exp Biol 2004;207:4559–72. 15579552 10.1242/jeb.01287

[13] Smith KE, Vanekeris LA, Linser PJ. Cloning and characterization of AgCA9, a novel alpha-carbonic anhydrase from *Anopheles gambiae* Giles sensu stricto (Diptera: Culicidae) larvae. J Exp Biol 2007;210:3919–30. 17981859 10.1242/jeb.008342

[14] a) Krungkrai J, Supuran CT. The alpha-carbonic anhydrase from the malaria parasite and its inhibition. Curr Pharm Des 2008;631–40. 18336308 10.2174/138161208783877901

[15] Pan P, Vermelho AB, Capaci Rodrigues G, et al Cloning, characterization, and sulfonamide and thiol inhibition studies of an alpha-carbonic anhydrase from *Trypanosoma cruzi*, the causative agent of chagas disease. J Med Chem 2013;56:1761–71. 23391336 10.1021/jm4000616

[16] Syrjänen L, Kuuslahti M, Tolvanen M, et al The β-carbonic anhydrase from the malaria mosquito *Anopheles gambiae* is highly inhibited by sulfonamides. Bioorg Med Chem 2015;23:2303–9. 25882523 10.1016/j.bmc.2015.03.081

[17] Altschul SF, Gish W, Miller W, et al Basic local alignment search tool. J Mol Biol 1990;215:403–10. 2231712 10.1016/S0022-2836(05)80360-2

[18] Hilvo M, Baranauskiene L, Salzano AM, et al Biochemical characterization of CA IX, one of the most active carbonic anhydrase isozymes. J Biol Chem 2008;283:27799–809. 18703501 10.1074/jbc.M800938200

[19] Khalifah RG. The carbon dioxide hydration activity of carbonic anhydrase. I. Stop-flow kinetic studies on the native human isoenzymes B and C. J Biol Chem 1971;246:2561–73. 4994926

[20] a) De Simone G, Supuran CT. (In)organic anions as carbonic anhydrase inhibitors. J Inorg Biochem 2012;111:117–29. 22192857 10.1016/j.jinorgbio.2011.11.017

[21] a) Scozzafava A, Briganti F, Mincione G, et al Carbonic anhydrase inhibitors: synthesis of water-soluble, aminoacyl/dipeptidyl sulfonamides possessing long-lasting intraocular pressure-lowering properties via the topical route. J Med Chem 1999;42:3690–700. 10479300 10.1021/jm9901879

[22] a) Scozzafava A, Menabuoni L, Mincione F, Supuran CT. Carbonic anhydrase inhibitors. A general approach for the preparation of water-soluble sulfonamides incorporating polyamino − polycarboxylate tails and of their metal complexes possessing long-lasting, topical intraocular pressure-lowering properties. J Med Chem 2002;45:1466–76. 11906288 10.1021/jm0108202

[23] a) Scozzafava A, Menabuoni L, Mincione F, et al Carbonic anhydrase inhibitors: perfluoroalkyl/aryl-substituted derivatives of aromatic/heterocyclic sulfonamides as topical intraocular pressure-lowering agents with prolonged duration of action. J Med Chem 2000;43:4542–51. 11087579 10.1021/jm000296j

[24] a) Syrjänen L, Tolvanen ME, Hilvo M, et al Characterization, bioinformatic analysis and dithiocarbamate inhibition studies of two new α-carbonic anhydrases, CAH1 and CAH2, from the fruit fly *Drosophila melanogaster*. Bioorg Med Chem 2013;21:1516–21. 22989910 10.1016/j.bmc.2012.08.046

[25] Soydan E, Güler A, Bıyık S, et al Carbonic anhydrase from *Apis mellifera*: purification and inhibition by pesticides. J Enzyme Inhib Med Chem 2017;32:47–50. 28090787 10.1080/14756366.2016.1232255 PMC6009862

[26] a) Carta F, Aggarwal M, Maresca A, et al Dithiocarbamates: a new class of carbonic anhydrase inhibitors. Crystallographic and kinetic investigations. Chem Commun (Camb) 2012;48:1868–70. 22218610 10.1039/c2cc16395k

[27] a) Supuran CT. Bortezomib inhibits bacterial and fungal β-carbonic anhydrases. Bioorg Med Chem 2016;24:4406–9. 27469982 10.1016/j.bmc.2016.07.035

